# From imaging to therapy: a novel photon-counting CT method for patient-specific image modeling and individualized atrial ablation strategies

**DOI:** 10.1007/s10840-026-02251-8

**Published:** 2026-02-12

**Authors:** Nico Erhard, Florian Englert, Marlene Siller, Keno Bressem, Martin Grosshauser, Jan Syväri, Miruna Popa, Hannah Kraft, Alex Tunsch Martinez, Madeleine Tydecks, Eva Koops, Dominic Dischel, Theresa Reiter, Marta Telishevska, Sarah Lengauer, Martin Hadamitzky, Gabriele Hessling, Isabel Deisenhofer, Fabian Bahlke

**Affiliations:** 1 Department of Electrophysiology, TUM University Hospital, German Heart Center, Munich, Germany; 2 Institute for Radiology and Nuclear Medicine, TUM University Hospital German Heart Center, Lazarettstr. 36, Munich, 80636 Germany; 3 Munich Data Science Institute, School of Natural Science, Technical University of Munich, Garching bei München, Germany

**Keywords:** Photon-counting detector computer tomography, Workflow integration, Individual ablation approach, Cardiac electrophysiology

## Abstract

**Introduction:**

In cardiac electrophysiology, preprocedural imaging has the potential to improve ablation strategies on an individual basis. Although clinical tools are increasingly being developed, data on practical workflows are limited. In this study, we sought to evaluate a high-resolution PCCT-based framework for individualized lesion design and digital modeling of ablation lines.

**Methods:**

In 198 patients, PCCT was performed before catheter ablation of atrial fibrillation (AF). Imaging data were semi-automatically segmented. Metrics such as left atrial wall thickness (LAWT) and epicardial adipose tissue (EAT) were extracted. Several ablation lesions were simulated, including linear lesions and circumferential pulmonary vein isolation (PVI) sets. LAWT along a standard ablation line was compared to individualized lines. For proof of concept, imaging-based decisions on lesion sets were applied to clinical cases.

**Results:**

In all 198 patients, the imaging resolution of the PCCT scan was sufficient for further semi-automated segmentation. Further analysis of wall thickness revealed a mean wall thickness in the left atrium of 1.57 ± 0.75 mm (range 0.1–8.25 mm; median 1.43 mm). Mean EAT thickness was 1.59 ± 2.11 mm (range 0–10.22 mm; median 1.01 mm). Using a custom-developed algorithm, ablation lesions could be simulated and measured for length, wall thickness distribution, and EAT.

**Conclusion:**

PCCT provides high-resolution structural imaging enabling individualized atrial modeling. Beyond visualizing atrial wall thickness and epicardial adipose tissue, this method forms the basis for an atrial twin, which allow virtual testing and optimization of ablation strategies. Personalized imaging may aid in creating continuous, durable lesions, minimizing the risk of reconnection and ablation failure.

**Graphical Abstract:**

**Figure:**
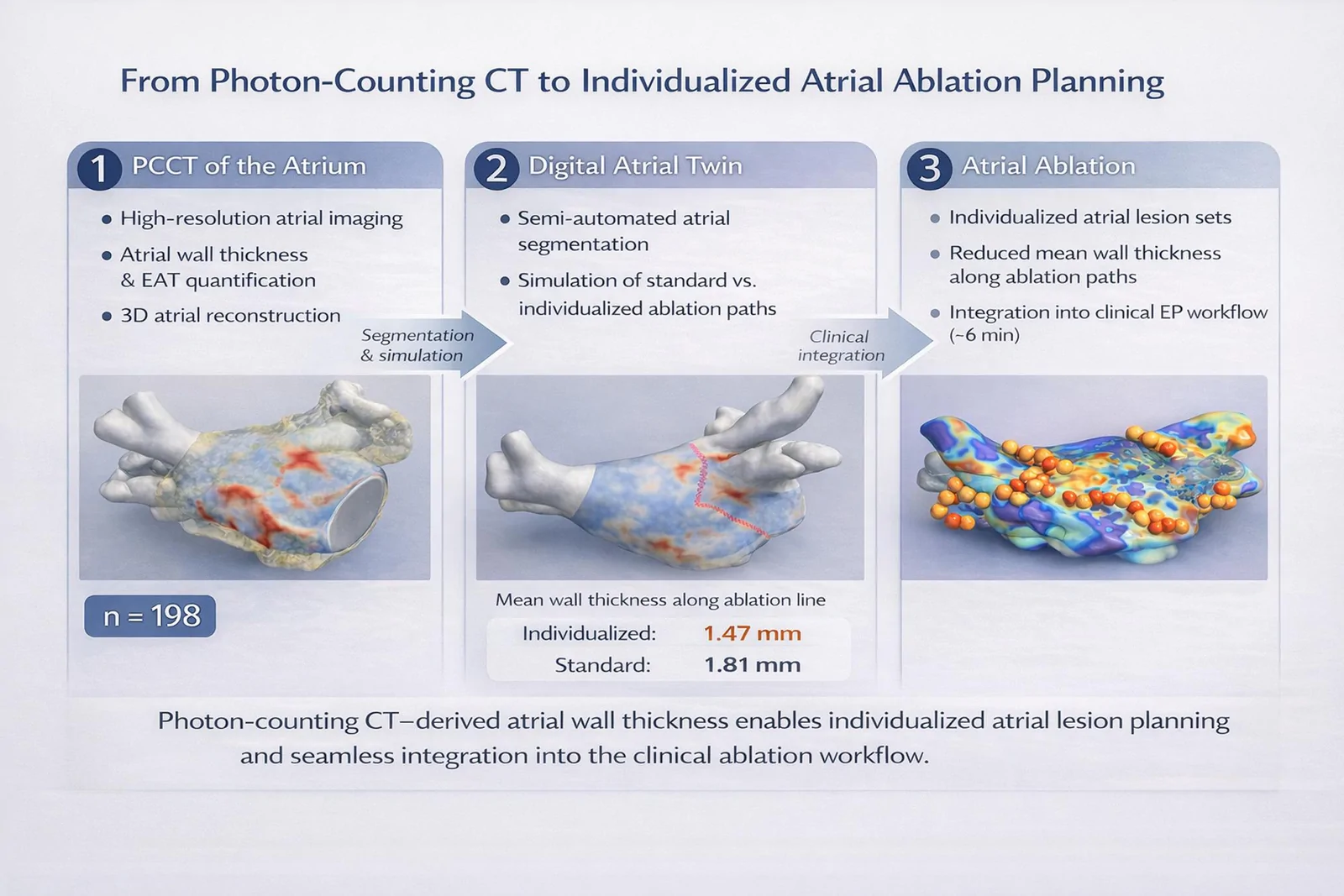

## Introduction

Left atrial catheter ablation has become well established to treat atrial fibrillation (AF), and technological advancements have made AF ablation safe and effective [1, 2]. Recent research is focused on ablation technologies with an increased interest in single-shot devices that aim to make AF ablation fast and reproducible, while providing an improved safety and efficacy profile compared to conventional ablation methods [3, 4]. Thus, focus has shifted away from standardized substrate mapping in AF patients to more generalized ablation approaches [5]. However, individual atrial substrate characteristics, such as scarring, left atrial wall thickness (LAWT), left atrial size, and epicardial adipose tissue (EAT), are essential when determining AF ablation success [6–8]. More complex atrial arrhythmias, such as persistent AF, atypical atrial flutter, or arrhythmias in adults with congenital heart disease, often require tailored lesion sets beyond single-shot ablation systems [9, 10].

Photon-counting detector computed tomography (PCCT) offers high spatial resolution and spectral imaging, allowing for detailed visualization of the atrial substrate [11, 12]. Recent advances in segmentation software allow semi-automated 3D reconstruction of cardiac anatomy for clinical and preclinicl use, supporting tailored ablation strategies and the development of patient-specific digital atrial twins [13–15].

We sought to establish a reproducible framework for analyzing segmented PCCT imaging data and for simulating customizable atrial lesion sets. This approach is particularly relevant in the context of atrial fibrillation and atrial flutter ablation, where even minor conduction gaps may critically compromise procedural success. As a secondary objective, we aimed to demonstrate that integrating pre-procedural imaging into the clinical workflow is feasible, rapid, and reliable.

## Methods

### Patient population

Imaging data of 198 prospectively included patients from 2022 to 2023 were exported for segmentation and further analysis. All patients were > 18 years old and underwent first-time catheter ablation for paroxysmal or persistent AF. Patients with prior left atrial catheter ablation, cardiac surgical history, congenital heart defects, or contraindications to computed tomography (CT) imaging were excluded from the study. PCCT imaging was performed 24 h before ablation in all patients to exclude left atrial thrombus formation. In addition, biatrial 3D reconstruction, including left- and right atrial anatomical modeling and assessment of LAWT and EAT, was performed.

Imaging data of 10 patients were imported into the 3D mapping system (NavX Ensite X, Abbott, Minneapolis, USA or CARTO3, Biosense Webster, Irvine, USA). Based on the LAWT, clinical decisions were made, and lesion sets were adapted.

The study was approved by the local ethics committee (approval 2022 − 243_1-S-NP, ethics committee of the Technical University of Munich) and was conducted following the Declaration of Helsinki. All patients gave informed consent.

### Photon-counting computer tomography

All patients underwent atrial PCCT imaging before AF ablation using the Siemens NAEOTOM Alpha CT scanner (Siemens Healthineers, Erlangen, Germany). PCCT provides enhanced spatial resolution and reduced image noise compared to conventional CT imaging while maintaining comparable radiation dosage. It enables precise visualization of critical structures, including the esophagus and EAT. The imaging protocol utilized a split-bolus contrast administration technique delivered through a cubital intravenous line. The contrast protocol consisted of three phases: an initial bolus of 50 mL iodine contrast agent (flow rate 4 mL/s), followed by a second bolus of 150 mL contrast agent mixed with mL 0.9% saline solution at a ratio of 30%/70%, at 3 mL/s, and a final 30 mL saline chaser at 3 mL/s. Scan timing was optimized using a test bolus technique. The protocol included two sequential scans: an arterial phase scan followed by a targeted left atrial appendage scan 60 s post-injection. Technical acquisition parameters included ECG-triggering (scan start at 70%, with patients positioned supine and arms elevated. Tube voltage was set to 140 kV, and tube current was automatically optimized for each patient using CareDose 4D (Siemens Healthineers, Erlangen, Germany). The scan was performed with a detector collimation of 144 mm × 0.4 mm, a pitch of 3.2, and a rotation time of 0.25s.

### Semiautomated 3-D atrial segmentation

PCCT atrial reconstructions were obtained using the inHEART platform (InHeart Medical, Bordeaux, France), a computer-based system designed to create a digital reconstruction of cardiac anatomy and substrate, including LAWT and EAT. Previous studies had demonstrated benefits regarding outcome and procedural time when integrating a ventricular reconstruction into the ablation workflow of ventricular tachycardias [13, 16, 17]. To the best of our knowledge, there is no data on using this imaging modality for atrial ablation. All atrial structures and the esophagus, aorta, and pulmonary arteries were segmented using proprietary convolutional neural networks. The LA wall was segmented as pixels with attenuation value above + 10 Hounsfield units in connection with the LA endocardium, after exclusion of adjacent structures (aorta, esophagus, RA, CS, pulmonary arteries, circumflex artery). Filtering operations were employed to obtain a smooth segmentation of the LA epicardium. The output was a LAWT map onto the LA endocardium geometry, expressed in mm. EAT was segmented as voxels with attenuation between − 1000 and − 10 Hounsfield Units located between the atria (LA and RA) and the pericardium, after subtraction of the esophagus and the lungs. The outputs were maps of EAT thickness onto the LA and RA endocardium geometries, expressed in millimeters (mm).

### Visualization of 3D-segmentations and simulation of potential ablation lesions

All exported 3D-segmentations were visualized and analyzed using ParaView 5.13.1 (Kitware Inc., Clifton Park, NY, USA). Several exemplary ablation lesions, such as circumferential PV isolation and different established ablation lines, were simulated to test the methodology. Anterior and lateral mitral lines (connecting the mitral annulus with the left superior PV, right superior PV, or right inferior PV) and several roof and posterior wall lines were analyzed regarding length, LAWT, and EAT.

The software was able to select all data points on a potential ablation line. Nevertheless, a dedicated algorithm was developed for the sequential listing of points along the ablation line, comprising three crucial steps. Using a k-nearest neighbors’ graph, each data point was connected to its k nearest neighbors. The algorithm defined the two points with the greatest distance between them as the start and endpoint of the line. After spline fitting to simulate the course of the ablation line, the distance covered was quantified to measure the line length. Lastly, the 3D curves were visualized for validation.

After successful simulation of ablation lines, individualized lesion sets of an anterior line connecting the anterior mitral annulus with the left superior pulmonary vein were created based on patient-specific patterns of wall thickening and thinning in 50 patients.

This framework may represent an initial step toward creating digital atrial twins, enabling virtual testing of ablation lines under patient-specific structural conditions.

Based on this validated framework, individualized anterior mitral lines were then generated in by adapting the lesion trajectory according to patient-specific patterns of atrial wall thickening and thinning. These simulations were intended to demonstrate technical feasibility of the approach.

### Catheter ablation

Ablation procedures were performed under monitored conscious sedation, using a regimen of midazolam, propofol, and fentanyl. Ultrasound-guided femoral access was performed, and three 8 F venous sheaths were inserted. High-density three-dimensional electroanatomical mapping of the left atrium was performed using either the EnSiteX and the Advisor HD Grid mapping catheter (Abbott, Minneapolis, USA) or the CARTO^®^ 3 and the Pentaray mapping catheter (Biosense Webster, Irvine, USA).

After high-density electro-anatomical mapping, the 3D inHEART model was merged using the pulmonary veins as anatomical landmarks. This allowed intraprocedural visualization of LAWT and EAT and surrounding anatomical structures such as the esophagus or phrenic nerve.

Wide antral circumferential point-by-point radiofrequency pulmonary vein isolation (PVI) was achieved using an irrigated-tip, contact force-sensing ablation catheter. This was done using a high-power short-duration (HPSD) protocol (65 W and 8–10 s for anterior PVI and roof, 65 W and 6 s for posterior PVI and posterior wall). For anterior linear lesions, LAWT information guided lesion placement, and ablation was performed using 50 W radiofrequency delivery for 30 s at each site.

### Statistical methods

Continuous variables were reported as mean (standard deviation), with differences between two independent groups assessed using Student’s t-tests. Binary and categorical variables were presented as absolute (n) and relative (%) frequencies, with group differences evaluated by chi-squared test or Fisher’s exact test. A two-sided p-value of < 0.05 was considered statistically significant. Data analysis was conducted using SPSS version 28.0 (IBM Corp, 2021) and R (R Foundation for Statistical Computing).

## Results

### Left atrial wall thickness and epicardial adipose tissue

**Fig. 1:**
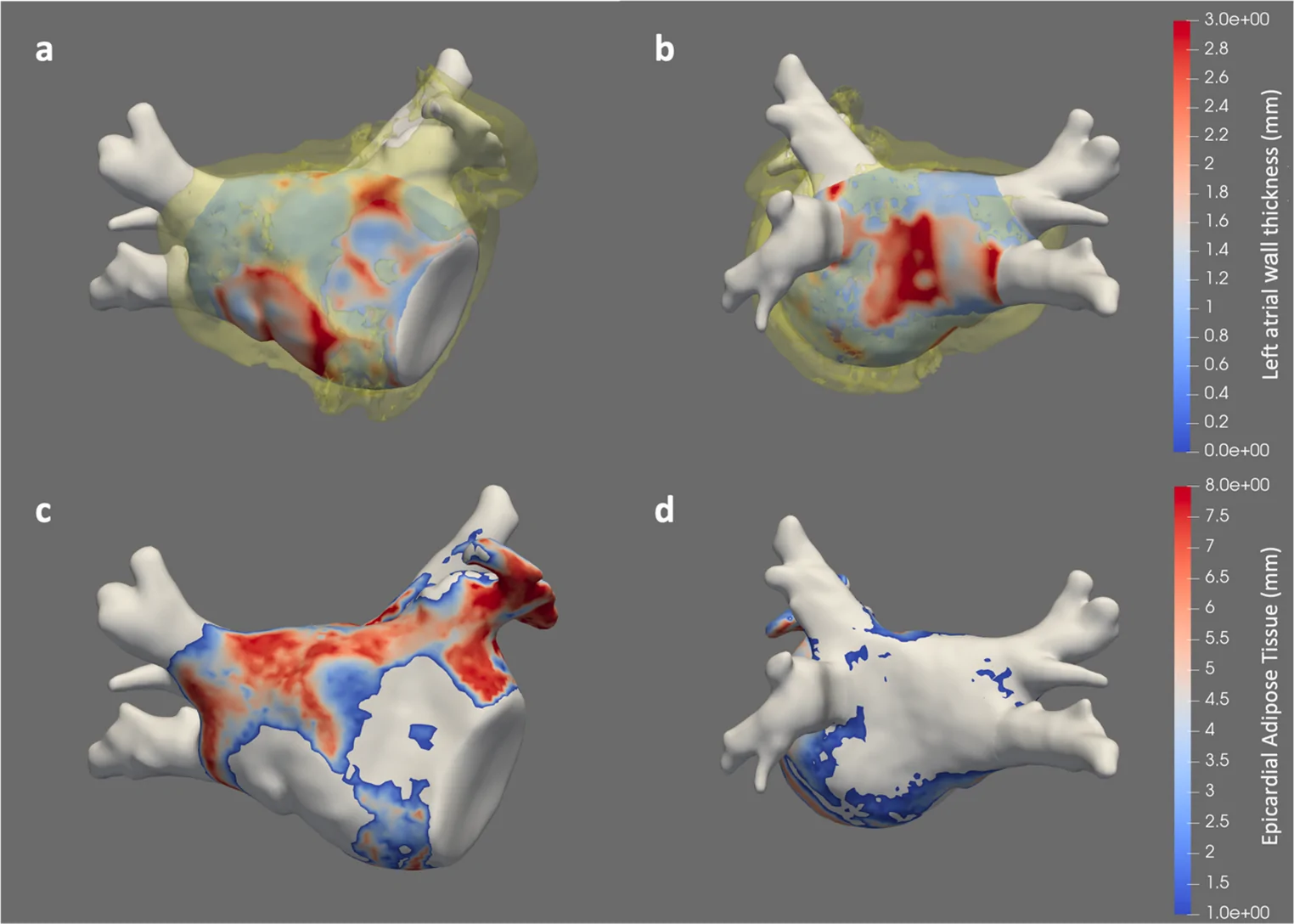
3D-visualization of left atrial segmentations. Panels a and b display left atrial wall thickness (LAWT) with overlaying epicardial adipose tissue (EAT) in yellow. Panels c and d show the thickness of the EAT alone. Panels are presented in anteroposterior view (**a** and **c**) and posteroanterior view (**b** and **d**)

Performing PCCT with the dedicated imaging protocol was completed in all patients, and image quality was adequate in all cases. Semi-automated 3D segmentation was successfully exported from the InHEART platform, and all maps were analyzed using ParaView software. Figure 1 shows an example of a left atrium with LAWT and EAT.

Including all data points of the LA body (*n* = 1035811, excluding pulmonary veins and the left atrial appendage), the mean LAWT was 1.57 ± 0.75 (range 0.1–8.25 mm; median 1.43 mm), and the mean EAT thickness was 1.59 ± 2.11 mm (range 0–10.22 mm; median 1.01 mm). 45.0% of the left atrium was free of EAT (466019/1035811 data points). A single LA body data set averaged 5231.4 data points. Mean LAWT at the posterior carina at the left and right PVs were 1.62 mm ± 0.61 and 1.69 mm ± 0.63, respectively.

### Simulation of ablation lesions

**Fig. 2:**
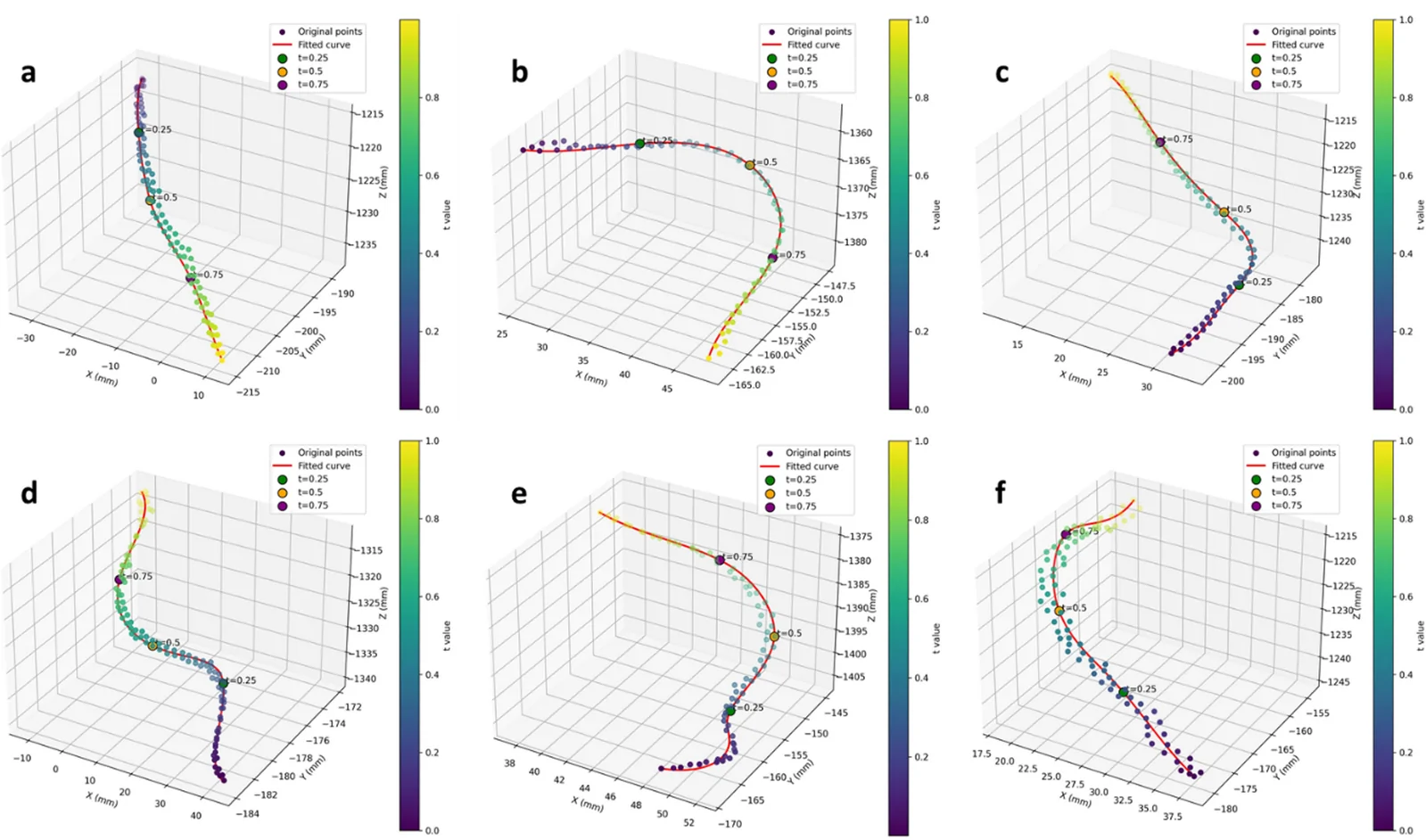
Simulation of ablation lines. Any desired ablation set was successfully simulated. Complex anatomies with curved lines and long distances were reproduced. After a successful simulation, further analysis of line length and differences in LAWT and EAT along the line was possible. Coordinates x-, y-, and z-axes, represent the three-dimensional coordinates (in millimeters) of the atrial geometry derived from the segmentation data.t is a normalized curve parameter (0–1) indicating relative position along the fitted 3D trajectory, with colour and markers highlighting corresponding locations (t = 0.25, 0.5, 0.75) along the path

**Fig. 3:**
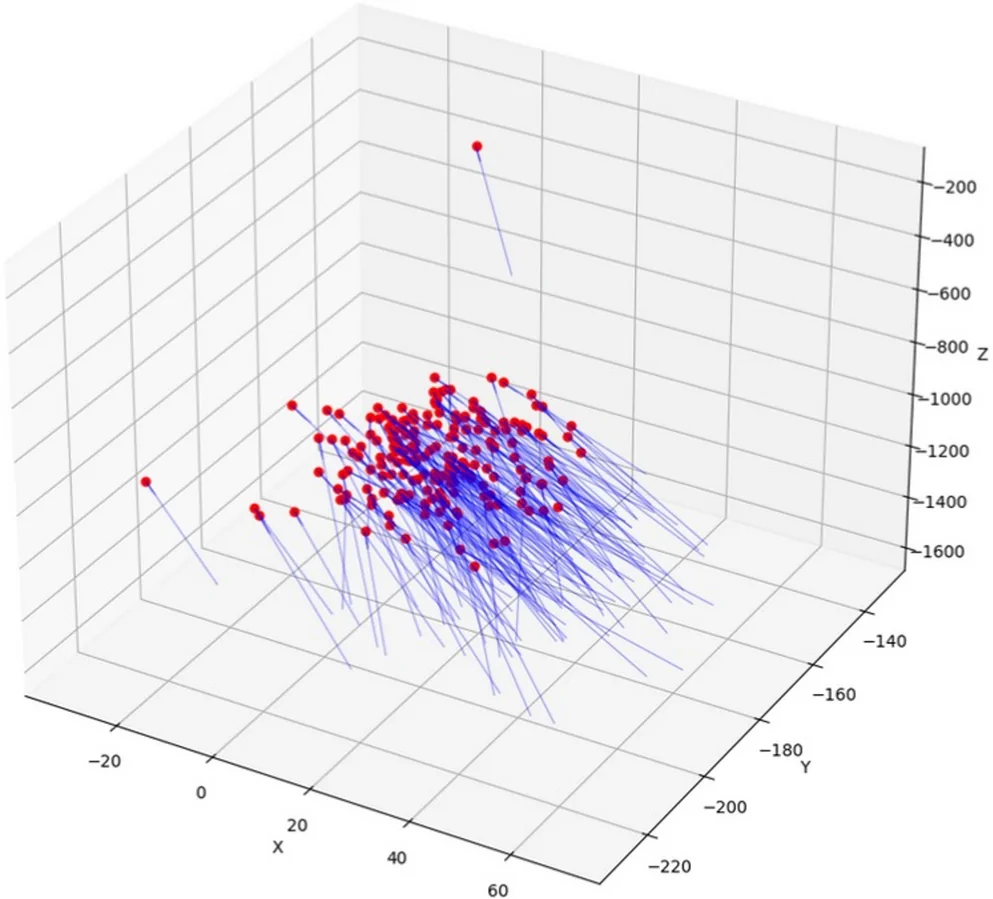
Secondary validation of line orientation. After initial simulation of linear lesions in the left atrium, the correct orientation of the start and endpoint was tested to validate proper data sorting. Each line represents a simulated linear lesion of a different patient. Visualizing and marking the endpoint with a red dot proved the correct orientation, and further analysis of linear lesions was possible. Coordinates x-, y-, and z-axes, represent the three-dimensional coordinates (in millimeters) of the atrial geometry derived from the segmentation data

After 3D-segmentation, it was feasible to imitate and further investigate ablation lines, including LAWT and EAT, in all patients using the custom-made alignment algorithm. Nine potential ablation lines were simulated in each patient. In all cases, analysis of length, LAWT, and EAT thickness was feasible. Figure 2 visualizes different shapes of ablation lesions, with data points properly sorted. Figure 3 shows the alignment of different anterior lines connecting the mitral annulus with the left superior pulmonary vein.

### Optimizing the lesion set based on wall thickness

**Fig. 4:**
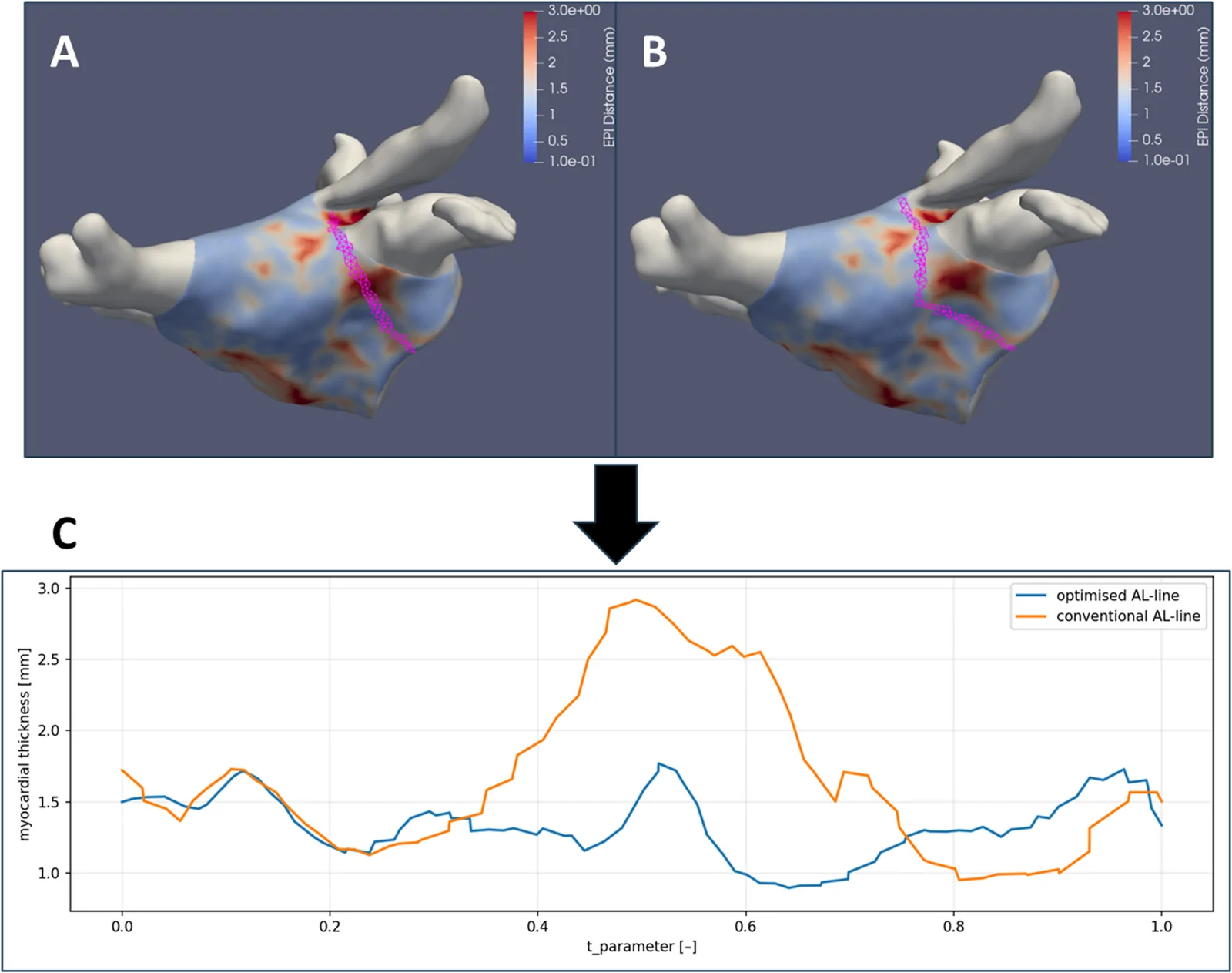
Exemplary lesion set for an anterior line connecting the left superior pulmonary vein with the mitral annulus. **A**: Conventional anterior line. **B**: Tailored anterior line avoiding thicker tissue. **C** Comparison of left atrial wall thickness along the exemplary lesion. Blue: optimised/tailored anterior line. Orange: conventional anterior line

**Fig. 5:**
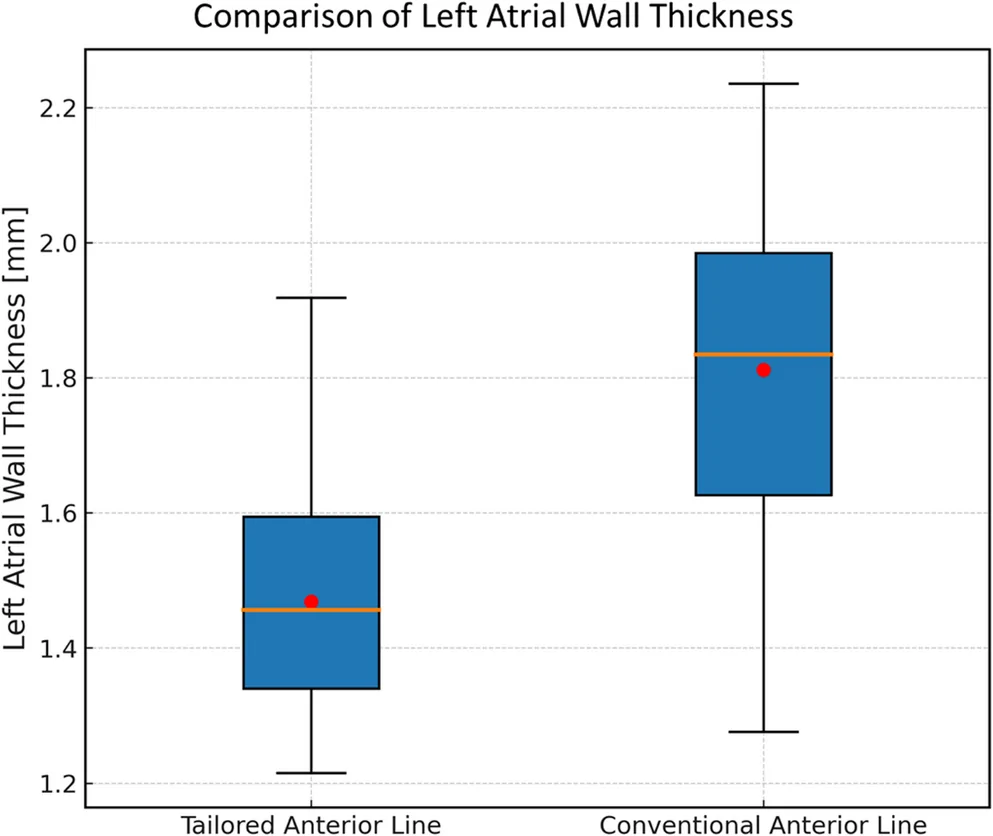
Comparison of the left atrial wall thickness (LAWT) in the tailored group and in the conventional group. Mean LAWT of each line was included in the analysis. Red dots visualize the mean value. LAWT was significantly lower in the tailored group (*p* < 0.001)

To quantify the potential reduction of LAWT along ablation lines, 50 individualized anterior lines were compared to 50 standard anterior lines connecting the mitral annulus and the left superior pulmonary vein **(**Fig. 4**)**. Mean LAWT along the standard anterior line was 1.81 ± 0.22 mm and along the individualized line 1.47 ± 0.16 mm (*p* < 0.001). Besides a lower mean LAWT, variability was reduced, as reflected by a lower standard deviation and interquartile range (0.36 mm vs. 0.25 mm, Fig. 5). The individualized lines were longer (line length 48.9 vs. 44.7 mm; *p* = 0.001).

### Workflow integration in clinical cases

In a subset of ten patients, PCCT-derived atrial reconstructions were successfully imported into the electroanatomic mapping system to demonstrate the feasibility of real-time implementation. The fusion of imaging and mapping data was consistently achievable and required 5.9 ± 3.7 min. Importantly, this step was performed in parallel with routine procedural preparations by a field technician, ensuring that it did not extend overall ablation duration. The mean total procedure time was 104.0 ± 16.1 min, which is comparable to procedures without image integration.

**Fig. 6:**
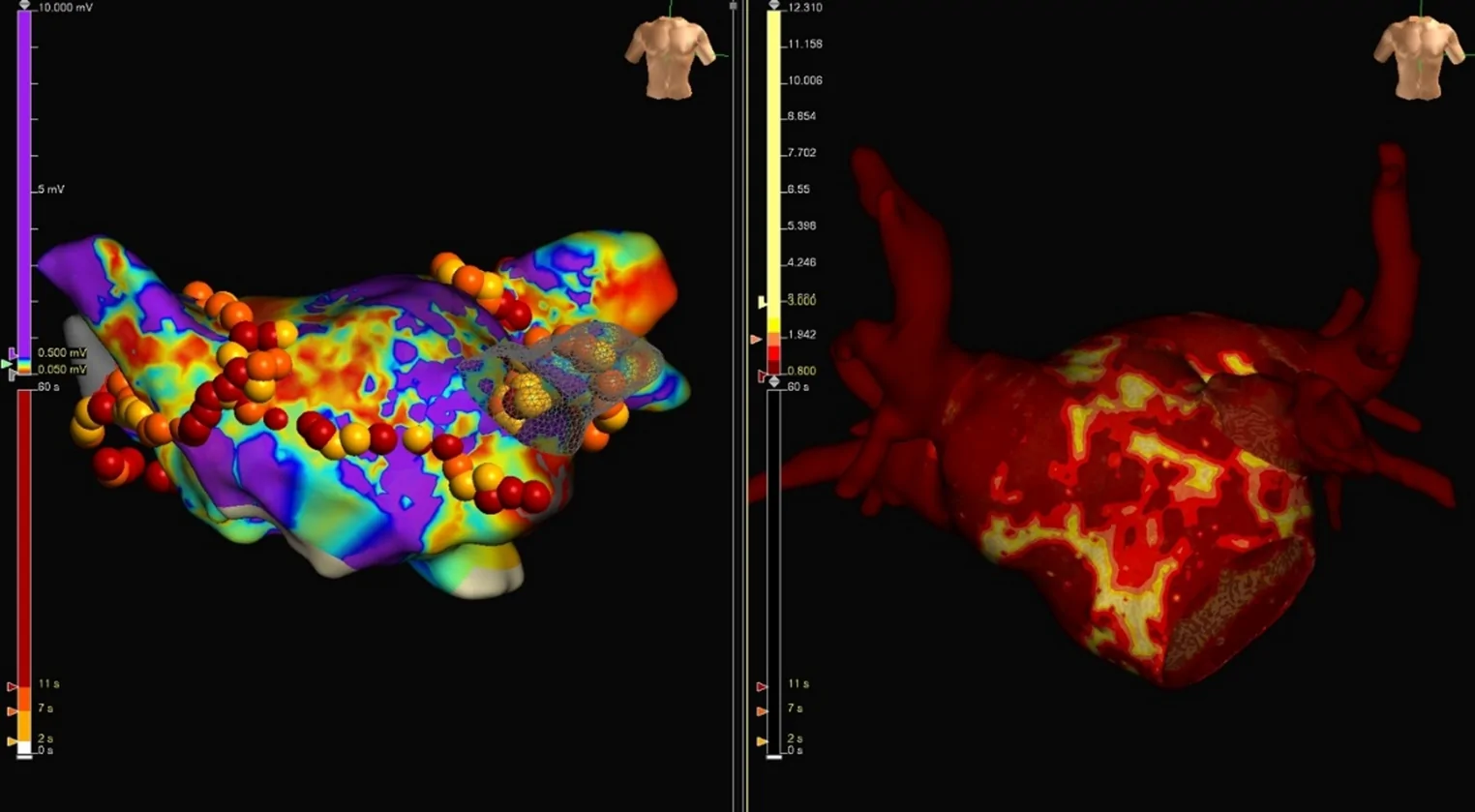
Integration of electroanatomical mapping and photon-counting CT segmentation. *Left*: High-density bipolar voltage map of the LA in AP view. Ablation points are displayed as red and yellow spheres. After successful pulmonary vein isolation, an anterior line was created connecting the mitral annulus with the RSPV. *Right*: Corresponding 3D-reconstruction derived from photon-counting CT with semi-automated segmentation of LAWT. Areas of increased LAWT are shown in yellow to white, thinner myocardium in dark red

Imaging information provided immediate access to patient-specific LAWT and EAT distribution before and during ablation. Beyond individualized pulmonary vein isolation, targeted linear lesions were applied if clinically indicated: one anterior line, one roof line, and one cavotricuspid isthmus line. In a representative case, the anterior line was adjusted toward an anteroseptal line, terminating at the right superior pulmonary vein to exploit a region of relative wall thinning, thereby avoiding a thicker myocardial region around the left atrial appendage. This example is depicted in Fig. 6. Acute PVI was achieved in 100% of patients. Also, all lines were tested and blocked in all procedures.

## Discussion

### Main findings

This study is among the first to demonstrate the feasibility of PCCT for atrial imaging. Beyond demonstrating feasibility, this study establishes a reproducible methodological framework for simulating and quantifying patient-specific ablation lesions. The focus lies on technical precision in lesion design, addressing the known limitation that even minimal conduction gaps may negate ablation success.

The main findings are as follows: First, semi-automated 3D segmentation of atrial imaging was possible in all 198 patients. Second, a specialized algorithm allowed simulation and quantitative analysis of potential ablation lesions, with personalized lesion sets effectively avoiding regions of wall thickening and reducing wall thickness. Third, integrating atrial 3D segmentation into the routine clinical workflow could be achieved within minutes. These results show the technical feasibility and possible clinical usefulness of image-guided, patient-specific ablation strategies.

### Feasibility of routine segmentation

Segmentation of PCCT data in a large cohort was consistently successful, with no patients excluded due to poor image quality or technical limitations. This highlights PCCT as a robust modality for atrial imaging, provided there are no contraindications to contrast agent administration. Beyond thrombus exclusion, PCCT provides quantitative parameters, such as LAWT and EAT, that cannot be derived from electroanatomical mapping alone. These imaging-derived metrics may serve as a foundation for optimized lesion set planning and for the reproducible, quantitative identification of arrhythmogenic substrate.

### Methodological insights

PCCT offers incremental advantages over traditional energy-integrating CT by providing 2–3 times higher spatial resolution, improved contrast-to-noise ratio, and inherent spectral capabilities [18]. 

In patients with atrial fibrillation, these features may improve preprocedural planning for catheter ablation and left atrial appendage occlusion by enabling more accurate delineation of left atrial anatomy, assessment of atrial wall characteristics, and detection of left atrial appendage thrombus, while maintaining high image quality at lower radiation doses [19].

These features allow for detailed visualization and quantitative assessment of atrial structures, including left atrial wall thickness and epicardial adipose tissue, at lower radiation and contrast doses. The resulting 3D reconstructions are especially suitable for segmentation and lesion modeling. PCCT appears to be a strong foundation for personalized ablation planning and future digital twin applications.

The study confirmed not only the ability to extract mean LAWT but also to simulate specific ablation lesions, such as pulmonary vein circles and linear lines. This method enables lesion design customized to each patient’s atrial anatomy, moving beyond trial-and-error ablation strategies. By creating patient-specific digital twins of the left and right atria, this approach enables virtual testing of ablation lesions before the procedure, potentially predicting lesion durability and improving personalized strategy choices.

Future work should characterize patterns of atrial wall thinning or thickening, as well as the distribution of EAT, within larger patient populations. Importantly, recent trials (SUPPRESS-AF, ERASE-AF [20, 21]) have shown inconsistent outcomes when substrate modification was guided solely by low-voltage areas. Differences in LAWT and EAT may represent additional determinants of arrhythmogenic remodeling that should be considered in AF ablation [22].

### Clinical implications

Durable lesion creation remains a central challenge in catheter ablation, despite the potential advantages of pulsed-field ablation (PFA) [23]. 

Information on wall thickness and EAT could improve outcomes by guiding lesion placement toward areas with greater transmurality while avoiding collateral injury. Our analysis showed that individualized line creation reduced the mean and median LAWT, as well as their variability (SD, IQR). From a clinical perspective, avoiding localized sites of excessive LAWT may be more relevant than reducing mean wall thickness, since a single gap or reconnection point can account for recurrence. Although the mean atrial wall thickness in our cohort was < 2 mm, achieving durable transmural lesions remains challenging in clinical practice. Factors such as catheter stability, cardiac and respiratory motion, convective cooling by blood flow, and local tissue characteristics may significantly affect effective energy delivery and lesion formation.

Emerging evidence further suggests that structural characteristics are closely associated with ablation outcome and may therefore contribute to improved procedural durability and long-term rhythm control when integrated into pre-procedural planning [24]. Incorporating detailed pre-procedural imaging may help to identify regions at risk for insufficient lesion formation, thereby enabling more durable lesion sets and potentially reducing future reconnection rates.

The integration of 3D segmentation into ablation procedures proved technically straightforward and time-efficient. On average, merging required 5.9 min and was performed simultaneously with routine tasks, thereby avoiding relevant procedural prolongation.

### Broader applicability

Although demonstrated here in left atrial ablation, this workflow has potential applications in other complex settings. In ablation of ventricular tachycardia, for example, preprocedural substrate quantification has already shown benefit in understanding arrhythmia mechanisms and guiding ablation strategies [11, 25]. Similarly, congenital heart disease or structurally complex atria may benefit from individualized lesion planning based on imaging-derived reconstructions.

### Outlook and role of pulsed field ablation

This study primarily demonstrated the feasibility of imaging-based lesion modeling. Future research could expand this framework, particularly by developing digital twin–based lesion simulations and their clinical implementation. Such advancements would enable personalized pre-procedural testing to optimize ablation lines, predict lesion durability, evaluate transmurality, and refine strategies. In subsequent studies, this approach should be correlated with procedural performance and clinical outcomes to evaluate its impact on ablation success and long-term rhythm control.

While pulsed field ablation (PFA) is a promising technology, it may not yet be suitable for all ablation scenarios. In particular, PFA applied near cardiac valves or within the ventricular myocardium has been associated with a low but clinically relevant risk of serious adverse events, including ventricular fibrillation related to coronary spasm [26]. Consequently, linear ablation strategies and ablation near coronary structures may continue to rely on radiofrequency ablation in the near term until the mechanisms underlying these events are better understood.

### Limitations

Image integration was perfomed at a single high-volume center, which may limit generalizability to other clinical environments with different ablation workflows or imaging expertise. While segmentation was technically feasible in all cases, image quality and reproducibility may vary across CT platforms and institutions, PCCT imaging is not widely accessible yet. Also, only a small cohort of ten patients underwent intraprocedural integration. The clinical utility of the workflow in larger and more complex populations remains to be established. Ablation outcomes as arrhythmia recurrence or lesion durability were not assessed in this study, and further prospective studies are needed to determine whether individualized lesion design translates into improved clinical endpoints.

The present study was designed as a proof of concept and therefore did not evaluate correlations between left atrial wall thickness and electroanatomical mapping parameters or assess clinical outcomes, which should be addressed in future, appropriately powered studies.

Finally, radiation exposure and contrast requirements of PCCT, should be carefully balanced against the potential benefits, particularly in younger patients or those with renal impairment.

## Conclusion

In this study PCCT-based 3D segmentation into AF ablation was practically possible in a routine clinical setting, provided quantitative data on atrial wall thickness and epicardial adipose tissue, and allowed for patient-specific customization of lesion sets.

The presented method forms the foundation for an atrial digital twin, enabling virtual testing and optimization of ablation strategies.

## Data Availability

No datasets were generated or analysed during the current study.
